# A COMPREHENSIVE DIGITAL SELF-CARE SUPPORT SYSTEM FOR CAREGIVERS AND PERSONS WITH DEMENTIA/MCI AND MCC

**DOI:** 10.1093/geroni/igae098.2963

**Published:** 2024-12-31

**Authors:** Priya Nambisan, Yura Lee, Julie Ellis, Murad Taani

**Affiliations:** University of Wisconsin Milwaukee, Milwaukee, Wisconsin, United States; University of Wisconsin-Milwaukee, Milwaukee, Wisconsin, United States; University of Wisconsin Milwaukee, Milwaukee, Wisconsin, United States; University of Wisconsin Milwaukee, Milwaukee, Wisconsin, United States

## Abstract

Research indicates that 95% of people with Alzheimer’s and other dementias also suffer from one or more other chronic conditions, increasing the risk of hospitalizations and complications such as contraindicated health care and drug interactions. Effective self-management practices are vital for maintaining cognitive function and preventing the onset of new chronic conditions among those with Multiple Chronic Conditions (MCC). However, managing MCC alongside cognitive impairment (CI) can make it very challenging for both patients and caregivers. Studies suggest that computer usage can enhance cognitive functioning and potentially delay further deterioration and digital apps are beneficial for self-care support. Despite this, there is a severe lack of technological applications that support those with CI and MCC and are often digitally excluded due to lack of understanding of their needs. We present the findings of a pilot test of a Comprehensive Digital Self-care Support System (CDSSS) known as myHESTIA. This system offers caregivers and persons with Alzheimer’s/Dementia a range of personalized tools including: 1) Health management tools: daily trackers for monitoring health conditions, reminders, to- do lists and educational resources; 2) Healing technologies: e.g. breathing exercises for relaxation, music therapy, learning activities like dancing or Yoga, and memory games and 3) Online social support forum: for connecting with others. This qualitative study aims to shed light on the perceived benefits of the system for both caregivers and persons with dementia/MCI, its utility in addressing unmet needs among those with CI, and its potential to improve the quality of life of caregivers.

